# Computer-Assisted Definition of the Inflammatory Infiltrates in Patients With Different Categories of Banff Kidney Allograft Rejection

**DOI:** 10.3389/fimmu.2019.02605

**Published:** 2019-11-08

**Authors:** Elena Aguado-Domínguez, Rocío Cabrera-Pérez, Alejandro Suarez-Benjumea, Cristina Abad-Molina, Antonio Núñez-Roldán, Isabel Aguilera

**Affiliations:** ^1^Department of Immunology, Instituto de Biomedicina de Sevilla, Hospital Universitario Virgen del Rocío, CSIC, Universidad de Sevilla, Seville, Spain; ^2^Department of Pathology, Instituto de Biomedicina de Sevilla, Hospital Universitario Virgen del Rocío, CSIC, Universidad de Sevilla, Seville, Spain; ^3^Department of Nephrology, Instituto de Biomedicina de Sevilla, Hospital Universitario Virgen del Rocío, CSIC, Universidad de Sevilla, Seville, Spain

**Keywords:** inflammatory infiltrate, newCAST, antibody-mediated rejection, borderline diagnostic, banff kidney classification, biopsy findings, M1 macrophages

## Abstract

Currently, the diagnosis of kidney allograft rejection relies on individual histological assessments made by expert pathologists according to the Banff classification. In this study, we applied new Computer-Assisted System Technology (newCAST™) by Visiopharm® with the aim of identifying and quantifying the immune cells in inflammatory infiltrates. We searched for distinctive cellular profiles that could be assigned to each rejection category of the Banff schema: antibody-mediated rejection (active and chronic active), borderline, T cell-mediated rejection (TCMR), and mixed rejection. This study was performed with 49 biopsy samples, 42 from patients with rejection and 7 from patients with clinical signs of dysfunction but an absence of histological findings of rejection. Plasma cells, B and T lymphocytes, natural killer cells, and macrophages, with a special focus on the M1 and M2 subsets, were studied. A major difference among the Banff rejection groups was in the total amount of cells/mm^2^ tissue. Principal component analysis identified some distinctive associations. The borderline category grouped with CD4^+^ lymphocytes and M1 macrophages, and active antibody-mediated rejection (aAMR) clustered with natural killer cells. Despite these findings, the search for characteristic profiles linked to the rejection types proved to be a very difficult task since the cellular composition varied significantly among individuals within the same diagnostic category. The results of this study will be analyzed from the perspective of reconciling the classic way of diagnosing rejection and the immune situation “*in situ*” at the time of diagnosis.

## Introduction

Clinical and subclinical rejections are the major causes of kidney allograft loss, and some types of rejection are difficult to diagnose and are detected late after the onset of the rejection process. Cellular analysis of the inflammation within a graft provides important information for a more precise diagnosis, but so far, this approach has not been incorporated into clinical practice. The first Banff classification for kidney allograft pathology, where experts came up with consensus diagnostic criteria, was published in 1993 and has been updated periodically since then (1). Some of the categories are well-characterized, but others are challenging. Among these categories, the process classified as borderline is not well-defined. At the first Banff meeting, this category was defined as mild focal interstitial inflammation and tubulitis with histological findings suggestive of a very mild acute rejection. At that time, several studies found that only a subset of patients diagnosed with borderline changes responded to antirejection therapy, raising questions regarding the heterogeneity of the processes included in the same group (2). Therefore, the question is whether the borderline category should be eliminated or at least redefined. With this purpose in mind, during the last Banff meeting in Barcelona, a series of histological and molecular studies were proposed to better organize this classification system (3).

An important aspect that has not been exhaustively addressed is the cellular composition of the inflammatory infiltrates in a biopsy at the time of the diagnosis of rejection. For instance, it has been demonstrated that macrophages have an important role as intermediaries between the allograft and T cells in the initiation of the alloimmune response (4). Macrophages are functionally heterogeneous and are mainly shaped by a variety of microenvironmental stimuli that drive polarization toward the M1 or M2 phenotype, both of which are related to specific functions. These two states are the two extremes of a phenotypic continuum dynamically influenced by the milieu (5). The aim of this study was to identify and quantify the main cell types present in the inflammatory infiltrates of patients with different diagnoses of rejection in accordance with the Banff criteria. For that purpose, we performed immunohistochemistry and double immunofluorescence staining with relevant cellular markers. For the analysis, we used a new Computer-Assisted Stereology method (newCAST™) with the aim of assigning cellular profiles to each rejection type included in the Banff classification, with special emphasis on the less-known borderline category.

Our hypothesis posits that not only the abundance but also the equilibrium among the components of the inflammatory infiltrate determine what forces prevail to drive the immune response toward one kind of rejection or another. This fragile equilibrium can be altered by many factors, from inadequate immunosuppression to signals associated with the innate immune response to infections. For this reason, detailed knowledge of the whole immune composition captured at the moment of diagnosis could certainly contribute to a more precise classification.

## Materials and Methods

### Patients

A cohort of 57 kidney transplant recipients was included in this study. The kidney transplantations were performed at Hospital Universitario Virgen del Rocío, Seville, Spain, between 2001 and 2014. Biopsy samples were always harvested in response to a clinical indication and not solely for the purpose of this study.

In total, 78 samples were analyzed, and all of them were anonymized and handled according to the ethical guidelines of the current Declaration of Helsinki, with the approval of the Hospital Universitario Virgen del Rocío ethical committee. Informed consent for publication was obtained from the patients. Seventeen of the samples did not show histological signs of rejection (NR), and 61 fulfilled the diagnostic criteria for one of the immune-mediated post-transplant pathologies: active antibody-mediated rejection (aAMR), chronic active antibody-mediated rejection (cAMR), T cell-mediated rejection (TCMR), mixed rejection (MR), or borderline (BL). Diagnoses were made by expert pathologists following the Banff criteria. All patients received steroid-based immunosuppression plus tacrolimus and mofetil mycophenolate (Table 1).

**Table 1:** Characteristics of patients included in the study.

**Characteristics**	**Total number of patients i-score analysis (*n* = 57)**	**Total number of patients newCAST technology analysis (*n* = 36)**
Mean recipient age	44 (12–73)	
Female	21 (37%)	
Male	36 (63%)	
First renal transplant	44 (77%)	
Second renal transplant	10 (18%)	
Third renal transplant	3 (5%)	
Donation after cardiac death	49 (86%)	
Donation in asystole	3 (5%)	
Living donor	5 (9%)	
Etiology of ESRD		
Nephroangiosclerosis	4 (7%)	
Diabetic nephropathy	1 (2%)	
Unknown kidney disease	20 (35%)	
Chronic tubuloint nephropathy	11 (19%)	
Glomerulonephritis	10 (18%)	
Systemic	1 (2%)	
Polycystic kidney disease	7 (12%)	
Congenital nephropathy	3 (5%)	
Baseline immunosuppression		
ST+ calc. inhib +MMF	50 (88%)	
ST+ calc. inhib + mTOR inhib	2 (3%)	
ST + mTOR inhib + MMF	5 (9%)	
Preformed DSA Class I/II	0	
*De novo* DSA Class I	2 (4%)	
*De novo* DSA Class II	7 (12%)	
Total number of biopsies	**78**	**49**
Non-rejection	**17** (22%)	**7** (14%)
Rejection	**61** (78%)	**42** (86%)
Active antibody-mediated	15 (19%)	11 (26%)
Chronic active antibody-mediated	18 (23%)	15 (36%)
Borderline	17 (22%)	10 (24%)
T cell-mediated	4 (5%)	1 (2%)
Mixed	7 (9%)	5 (12%)

*ESRD, end-stage renal disease; ST, steroids (prednisone or methylprednisolone); calc. inhib, calcineurin inhibitors (cyclosporine or tacrolimus); MMF, mofetil mycophenolate; mTOR inhib, mTOR inhibitors (sirolimus or everolimus); DSA, HLA donor-specific antibodies. Bold highligts the total number of biopsies included on each part of the study*.

### Cell Markers

Cells were immunohistochemically identified with the following markers: CD20 for B lymphocytes, CD138 for plasma cells, CD4 or CD8 for T lymphocytes, CD56 for NK cells, and FoxP3 for T regulatory cells. Macrophages were immunofluorescence-stained with an anti-human CD68 antibody and pSTAT1 or cMAF, which are transcription factors localized in the nuclei of M1 and M2 macrophages, respectively (6). All antibodies used and their respective dilutions are summarized in the Supplementary Table 1.

### Immunohistochemistry

Serial sections of three-micrometer-thick formalin-fixed, paraffin-embedded tissue sections were handled as described (7). Incubation conditions for the primary antibodies are included in Supplementary Table 1. EnVision™ system-horseradish peroxidase (HRP)-labeled polymer anti-mouse (Dako, ref. K4000) for mouse primary antibodies and EnVision™ system-HRP-labeled polymer anti-rabbit (Dako, ref. K4011) for rabbit primary antibodies were incubated for 40 min at room temperature.

For the negative controls, the same protocols were followed, but the primary antibody was omitted to avoid non-specific signals.

### Immunofluorescence

Macrophages and the polarized subpopulations M1 and M2 were identified by immunofluorescence staining with the primary anti-pSTAT1 and anti-cMAF rabbit antibodies, respectively. An Alexa Fluor® 568-conjugated goat anti-rabbit antibody (Life Technologies, ref. A11011) in combination with anti-CD68 primary antibody and then an Alexa Fluor® 488-conjugated goat anti-mouse (Life Technologies, ref. A11001) were incubated with the sections at a 1:500 dilution for 30 min at RT.

### New Computer-Assisted System Technology

**Figure 1:**
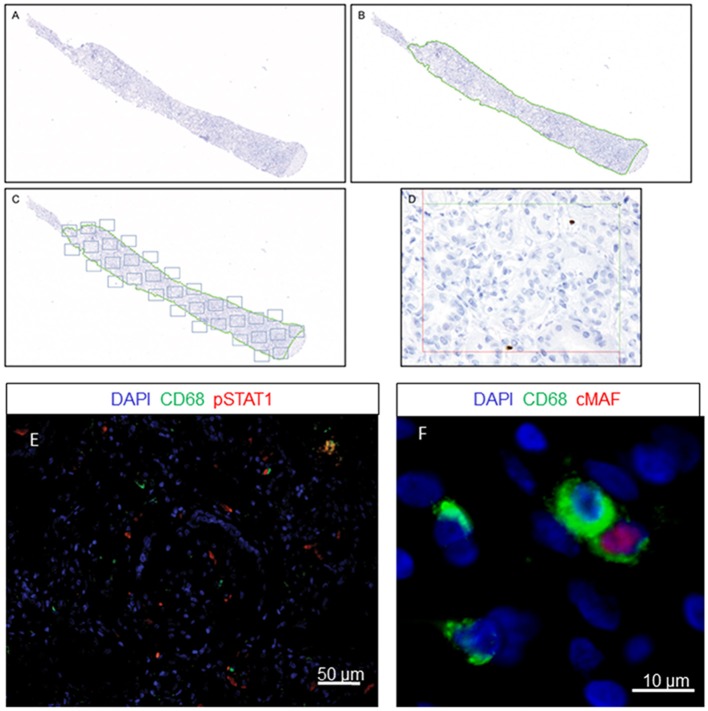
Quantification method using newCAST™ Visiopharm® software. Representative images showing super image capture of a biopsy sample **(A)**; manual outlining of the region of interest (ROI) excluding non-clinically relevant areas, indicated by the green dotted line **(B)**; unbiased random sampling **(C)**; detailed cell quantification at 400x magnification **(D)**. Double-immunofluorescence staining for CD68 and pSTAT1 **(E)** or cMAF **(F)**.

The renal cortex was exhaustively evaluated by employing a stereological method that avoids observer subjectivity using new Computer-Assisted System Technology (newCAST™) by Visiopharm®. The newCAST™ stereology system was previously used in other transplant pathologies (7). To our knowledge, this work is the first application in human kidney transplantation. The mean sample tool was used following Visiopharm® protocols and recommendations (http://www.visiopharm.com); a super image of the whole slide was captured at the beginning (Figure 1A). The operator then manually excluded the medulla and other non-relevant areas in terms of clinical significance. The tissue sample was outlined as the region of interest (ROI) and indicated by a green dotted line (Figure 1B). The computer-assisted software selected areas within the ROI by systematic random sampling (gray squares) and analyzed them in a 400x magnification field of view (Figures 1C,D). A total of 30% of the ROI was assessed by computer-assisted random sampling. This approach identified the optimal percentage accepted to achieve statistically sufficient precision in cell quantification. Double-immunofluorescence staining identification was supported by ImageJ software, where double-positive CD68^+^/pSTAT1^+^ and CD68^+^/cMAF^+^ cells were identified in the ROI (Figures 1E,F). The quantification of every cell type included in the study is represented as cells/mm^2^.

### Statistical Analysis

The normality of the data was tested using the Kolmogorov-Smirnov assay. Due to non-normally distributed data, the median with interquartile range (IQR) was represented unless otherwise indicated. Data were analyzed using Mann-Whitney and Kruskal-Wallis tests for non-parametric variables. A chi-square test for categorical variables was used. The results were considered statistically significant when *p* values were < 0.05. GraphPad Prism® Software (La Jolla, California, USA) was used for representation of the results.

## Results

Among 78 biopsies corresponding to 57 kidney transplants, 61 fulfilled the diagnostic criteria for different rejection categories and were distributed as follows: 15 aAMR biopsies, 18 cAMR biopsies, 17 BL biopsies, 4 TCMR biopsies, and 7 MR biopsies. The remaining 17 biopsies, performed due to renal dysfunction, did not show histological signs of rejection and were used as the non-rejection group (NR). All these biopsies were classified with the Banff schema, but only 49 of them were analyzed with newCAST™ due to a shortage of tissue samples. Demographic data are detailed in Table 1.

### Analysis of Graft Inflammation With newCAST™

**Figure 2:**
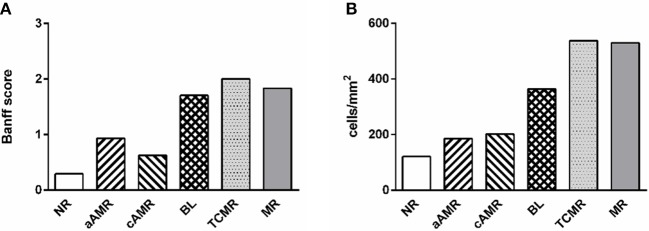
Comparison of two approaches to diagnosing rejection. Mean value of inflammation in every diagnostic category measured by the Banff score from 0 to 3 **(A)** and with the computer-assisted quantification technique performed in this study **(B)**.

We identified relevant cell types present in the interstitium and glomeruli of the renal cortex. Inflammatory cells were negligible in the glomeruli; therefore, the data presented refer only to the interstitium. Notably, the mean values of the total number of cells, including every type, correlated well with the mean inflammation profile of the total Banff score for each rejection group, confirming the strength of our method (Figure 2). The scores for the parameters included as diagnostic criteria by the Banff Working Group in the biopsies included in our study are summarized in the Supplementary Figure 1.

### Phenotypes of Infiltrating Cells in Different Categories of Banff Kidney Allograft Rejection

**Figure 3:**
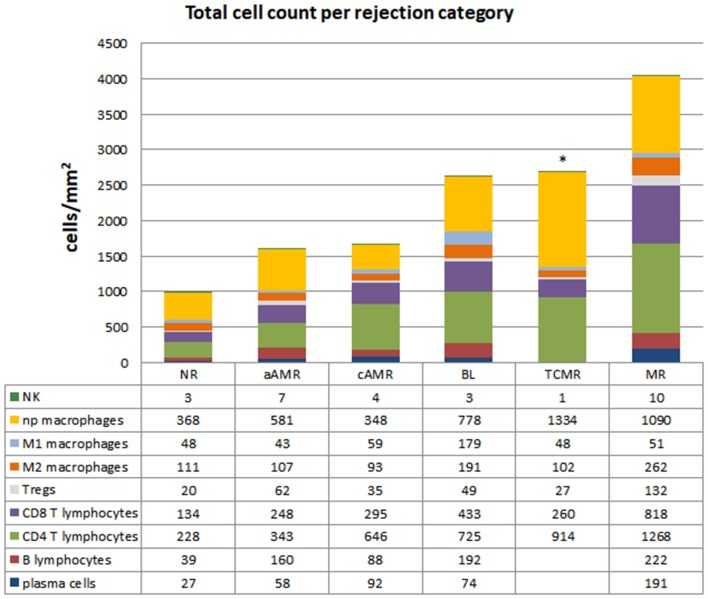
Total cell counts in diagnostic biopsies of rejection. Cell quantification of the inflammatory infiltrates showing cell type composition. The numbers for each cell type represent the mean values obtained from a group of patients within each rejection category. CD20^+^ B lymphocytes, CD138^+^ plasma cells, CD4^+^ T lymphocytes, CD8^+^ T lymphocytes, T regulatory cells FoxP3^+^, CD68^+^ (distributed as non-polarized, M1 and M2), and CD56^+^ natural killer cells were assessed. ^*^The data for B lymphocytes and plasma cells are missing in the TCMR group due to the low number of tissue samples. The mean numbers of each cell type/mm^2^ tissue are shown below each group.

The characteristics of the biopsies included in this study are detailed in Table 2. The quantification of the cells in the infiltrates of the different Banff diagnostic category groups and the non-rejection group is shown in Figure 3. The data are represented as the mean number of each type of immune cell/mm^2^ of tissue. The infiltrates in the NR biopsies had the lowest number of cells (975 cells/mm^2^), followed by those in the aAMR and cAMR biopsies, with 1,506 cells/mm^2^ and 1,598 cells/mm^2^, respectively. The borderline category had 2,694 cells/mm^2^, a value that was almost double the value for the AMR categories but was still lower than that of the MR category, which had the highest value of all, 4,032 cells/mm^2^.

**Table 2:** Description of the biopsies included in the study.

**Banff category** **patient code**	**Biopsy (time after Tx)**	**Diagnosis**	**HLA-DSA**	**C4d**
**Normal Biopsy**				
NR-1	8 d	Non-rejection	Neg	Neg
NR-2	12 d	Non-rejection	Neg	Neg
NR-3	30 d	Non-rejection	Neg	Neg
NR-4	24 d	Non-rejection	Neg	Neg
NR-5	18 d	Non-rejection	Neg	Neg
NR-6	4 y	Non-rejection	Neg	Neg
NR-7	4 m	Non-rejection	Neg	Neg
**Active Amr**				
aAMR-1 (B1)	9 d	aAMR grade I	Class I	+
(B2)	16 d	aAMR grade II	Class I	+
aAMR-2 (B1)	19 d	aAMR grade I	Class I	+
(B2)	5 m	aAMR grade II	Class I	+
aAMR-3	8 y	aAMR grade II	Class I	+
aAMR-4	12 d	aAMR grade II	Class I	+
aAMR-5	25 d	aAMR grade I	Class I	+
aAMR-6	3 m	aAMR grade II	Neg	+
aAMR-7	19 y	aAMR grade II	Class II	+
aAMR-8	11 y	aAMR grade II	Class II	+
aAMR-9	5 y	aAMR grade II	Non-HLA	+
**Chronic Amr**				
cAMR-1	14 y	TxG	Neg	Neg
cAMR-2	9 y	TxG	Neg	Neg
cAMR-3	6 y	TxG	Non-HLA	+
cAMR-4 (B1)	13 y	TxG	Class II	Neg
(B2)	14 y	TxG	Class II	Neg
cAMR-5	12 y	TxG	Neg	Neg
cAMR-6	6 y	TxG	Neg	Neg
cAMR-7	6 y	TxG	Class II	Neg
cAMR-8	26 y	TxG	Neg	Neg
cAMR-9	11 y	TxG	Neg	Neg
cAMR-10	16 m	TxG	Class II	Neg
cAMR-11	10 y	TxG	Neg	Neg
cAMR-12	21 m	TxG	Class II	Neg
cAMR-13	10 y	cAMR grade III	Class I	+
cAMR-14	5 y	cAMR grade III	Class II	+
**Borderline**				
BL-1	6 y	Borderline	Neg	Neg
BL-2	8 d	Borderline	Neg	Neg
BL-3	1 y	Borderline	Neg	Neg
BL-4	4 y	Borderline	Neg	Neg
BL-5	30 d	Borderline	Neg	Neg
BL-6 (B1)	40 d	Borderline	Neg	Neg
(B2)	60 d	Borderline	Neg	Neg
BL-7	3 m	Borderline	Neg	Neg
BL-8	8 m	Borderline	Neg	Neg
BL-9	18 d	Borderline	Neg	Neg
**Tcmr**				
TCMR-1	5 d	TCMR IIB	Neg	Neg
**Mixed Rejection**				
MR-1	2 y	aAMR II/BL	Class I/II	+
MR-2	90 d	aAMR II/TCMR IIA	Neg	+
MR-3	6 y	aAMR II/TCMR IIB//TxG	Class I/II	+
MR-4	30 d	aAMR I/BL	Neg	+
MR-5	1 y	aAMR I/BL	Class II	+

*NR, non-rejection; aAMR, active antibody-mediated rejection; cAMR, chronic active antibody-mediated rejection; BL, Borderline; MR, mix rejection; TxG, transplant glomerulopathy; TCMR, T cell-mediated rejection; d, days; m, months; y, years; DSA, donor-specific HLA antibodies*.

**Figure 4:**
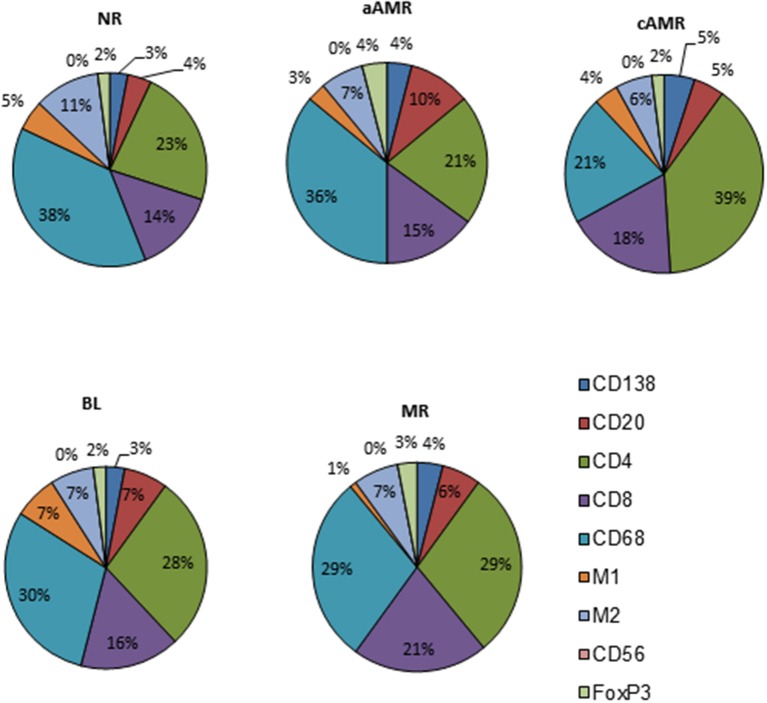
Cellular profiles of the inflammatory infiltrates in diagnostic biopsies of four rejection types included in the Banff classification and a non-rejection type.

With the purpose of defining profiles that could uncover specific features among the diagnostic categories of the Banff classification, we performed a close analysis of the cellular composition in each of the rejection type groups and the non-rejection group (Figure 4, Supplementary Table 2). The main observation was the predominance of T lymphocytes and macrophages, covering 86–93% of the infiltrates, with CD4 as the most abundant T-cell population in all the groups studied. After assessing these two populations, the rest of the cell types assayed comprised very small proportions of the infiltrates (7–14%). A detailed description of each profile is given in the Supplementary Figures 2–6.

### Significant Differences Among the Banff Rejection Groups

**Figure 5:**
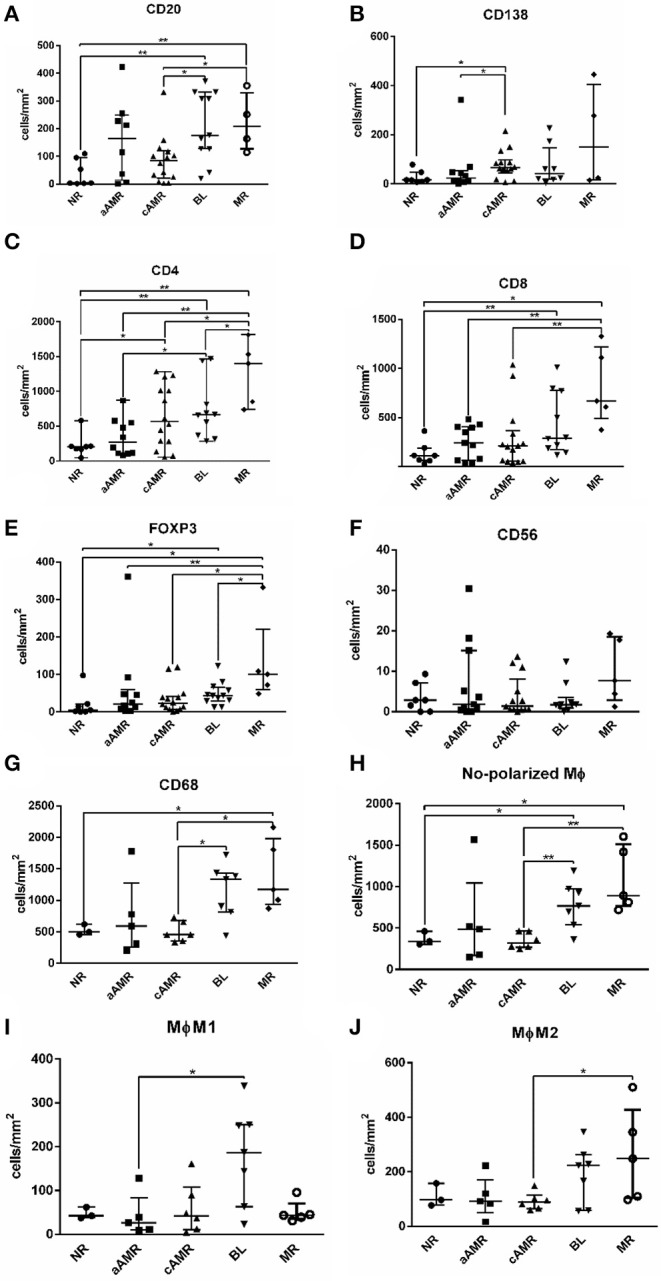
Comparison of the number of cells/mm^2^ tissue among Banff rejection categories. CD20^+^ B lymphocytes **(A)**, CD138^+^ plasma cells **(B)**, CD4^+^ T lymphocytes **(C)**, CD8^+^ T lymphocytes **(D)**, FoxP3^+^ T regulatory cells **(E)**, CD56^+^ natural killer cells **(F)**, total CD68^+^ ϕ **(G)**, non-polarized ϕ **(H)**, M1 ϕ **(I)**, and M2 ϕ **(J)**. The results are presented as the median with the interquartile range. ^*^p < 0.05, ^**^p < 0.01. Values plotted as individual black dots represent outliers that fell outside of the upper whisker (the 75th percentile) or the lower whisker (the 25th percentile).

The total number of cells identified by each cell marker was compared among the 5 groups included in the study, and the results obtained are shown in Figure 5. TCMR is a very uncommon diagnosis at our hospital and was excluded from this analysis. Most of the markers assayed seemed useful for comparing rejection groups, except CD56^+^ natural killer (NK) cells, which were not discriminatory for any of the rejection groups (Figure 5F). The first thing that caught our attention was the lack of significant differences between NR and aAMR for all the cell types analyzed. Curiously, the two groups with a strong humoral component, aAMR and cAMR, showed a significant difference in the amount of CD138^+^ plasma cells (*p* < 0.05) (Figure 5B). In the BL group, where an important role for T cells has been suggested, significant differences were seen from the aAMR and MR groups in terms of the amount of CD4^+^ cells and from the MR group in terms of the amount of FoxP3^+^ cells, while the amount of CD8^+^ cells did not distinguish the BL group from any of the other groups (Figures 5C–E). An additional discriminating feature between BL and cAMR was the substantial presence of CD20^+^ B lymphocytes (*p* < 0.05) (Figure 5A). MR showed augmented numbers of all three T lymphocyte types tested, CD4^+^, CD8^+^, and FoxP3^+^ (*p* < 0.01). Similar to the contrast between BL and cAMR, CD20^+^ B lymphocytes were a distinguishing feature between MR and cAMR (*p* < 0.05).

Regarding macrophages, we first compared the total amount of macrophages and then the non-polarized, M1, and M2 subsets separately among the rejection groups. The first analysis showed statistically significant differences between cAMR and BL and between cAMR and MR (Figure 5G) that persisted when the analysis was performed with the non-polarized population (Figure 5H). However, the most interesting results appeared when the analysis was performed with the polarized populations; the first finding was the strong presence of proinflammatory M1 macrophages in BL, which exhibited higher M1 macrophage infiltration than the rest of the groups, although this difference only reached statistical significance between BL and aAMR (Figure 5I). Since BL is not a well-characterized entity, the presence of such a high number of M1 macrophages could be a specific marker for this pathology that deserves further study. The second finding was the abundance of M2 macrophages in MR, although this difference achieved statistical significance only between MR and cAMR (Figure 5J).

### Differences Between TCMR and BL

At the time that the Banff 2015 meeting was held, there was a working group dedicated to improving the diagnosis of “pure” TCMR, with the reintroduction of the i-IFTA score as one of the aspects under consideration (8). The working group was also evaluating the possible elimination of BL from the Banff classification. The next update published in 2017 maintained both TCMR and BL, not as separate categories but as one group with overlapping features. We found that the amount of T lymphocytes/mm^2^ tissue in TCMR and BL was exactly the same (1,201 vs. 1,207, respectively) but that the composition differed (914 vs. 725 for CD4^+^; 260 vs. 433 for CD8^+^; and 27 vs. 49 for FoxP3, respectively). Regarding macrophages, BL showed 4 times more M1 cells (179 vs. 48, respectively) and almost double the number of M2 macrophages (191 vs. 102, respectively) compared to TCMR (see Figure 3).

### Cell Composition in Follow-Up Biopsies After Therapy

**Figure 6:**
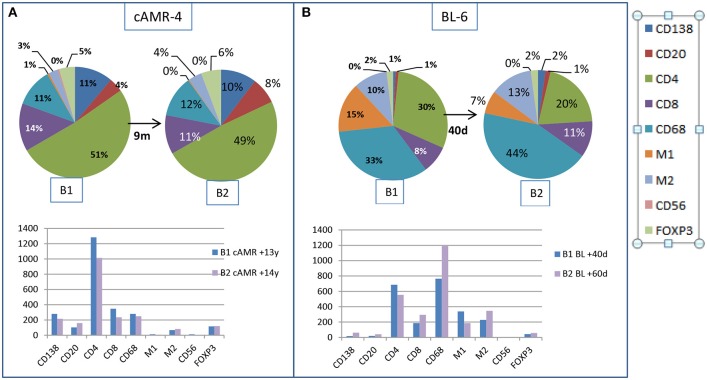
Changes in the interstitial infiltrates over time. Comparison of the amount of cells between two consecutive biopsies from a patient with cAMR **(A)** and a patient with BL diagnosis **(B)**.

We had the opportunity to compare changes in the cellular composition after rejection therapy in well-characterized consecutive biopsies from two patients. The first patient, cAMR-4, was diagnosed 13 years after transplantation, and a second biopsy was performed 9 months after diagnosis. There were no remarkable changes in cell composition, with the exception of B lymphocytes, which increased from 4 to 8% in the second biopsy. The number of cells decreased in all cellular types, especially CD4^+^ and CD8^+^, except B lymphocytes and M2 cells (Figure 6A). The second patient, BL-6, was diagnosed with BL 40 days after transplantation and had a second biopsy performed 20 days after diagnosis. In the second biopsy, we observed two important changes. Although the proportion of total macrophages was essentially unchanged, 58% vs. 64%, M1 cells diminished from 15 to 7% in favor of M2 cells, which increased from 10 to 13%, and especially in favor of non-polarized macrophages, which increased from 33 to 44%. The second remarkable change was the loss of 1/3 of the CD4^+^ T lymphocytes, whose proportion decreased from 30 to 20%. CD8^+^ cells experienced an increase from 8 to 11%. Tregs, PCs, and B lymphocytes did not change substantially and maintained a low number of cells in both biopsies. If we looked at the total number of cells, CD4^+^ and M1 cell numbers decreased in the second biopsy while the numbers of non-polarized and M2 macrophages was augmented. The number of CD8^+^ cells increased, and PCs, B lymphocytes, NK cells, and Tregs remained practically unchanged, but these cells comprised a very small proportion of the infiltrates (Figure 6B).

### Global Analysis of the Immune Response in the Banff Categories of Rejection

**Figure 7:**
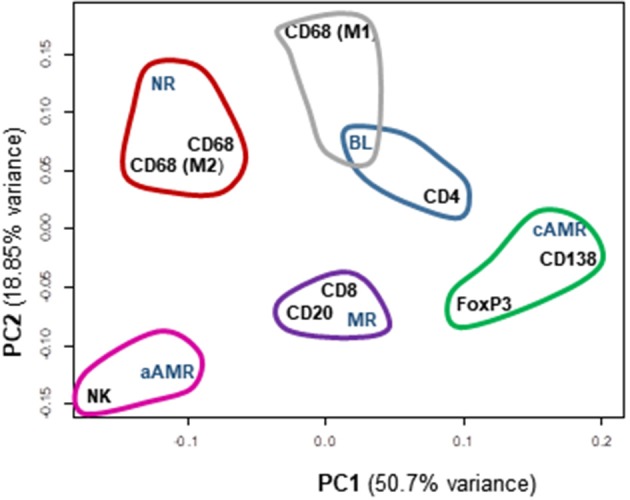
Principal component analysis (PCA) scatterplot of cellular types of the inflammatory infiltrates among different Banff rejection categories. Clustering of the most similar entities among the rejection categories and all the cellular components is shown. PC1 and PC2 are the two principal component axes. NR, non-rejection; BL, borderline; aAMR, active antibody-mediated rejection; cAMR, chronic active antibody-mediated rejection; MR, mixed rejection.

When taking all cellular types into account, the rejection category groups and the control group segregated quite well. A multidimensional scaling plot revealed the clustering of cellular types with certain diagnostic entities, as shown in Figure 7. PC2 had large positive associations with NR and BL and negative associations with aAMR, MR, and cAMR; therefore, PC2 differentiates the NR and BL categories (upper) from the aAMR, MR, and cAMR categories (lower), suggesting that the correlations between NR and BL and among aAMR, MR, and cAMR are stronger than other correlations, probably because of the participation of a true rejection process in terms of inflammation in aAMR, MR, and cAMR. PC1 did not show any association with MR or BL but had positive correlations with NR, aAMR, and cAMR.

A clustering analysis was performed based on the entities that were placed close to one another. This analysis demonstrated that differences exist between the 5 groups included in the study. Some clusters and trends could be identified: BL clustered with CD4^+^ cells and M1 macrophages; cAMR clustered with CD138^+^, FoxP3^+^, and CD4^+^ cells; MR clustered with CD20^+^ and CD8^+^; and finally, aAMR clearly clustered with NK cells. Although the number of NK cells in the aAMR biopsies did not show any significant difference from that in the other biopsies when the mean values were compared, in this analysis, we were able to confirm that NK cells and aAMR cluster together and that both entities show more similarities between them than with any other category of rejection. Macrophages in general and the M2 subset clustered with NR and not with any other category, whereas the M1 subset appeared closest to BL, and, consistent with the results of this study, we believe that M1 cells should also be included in this cluster together with CD4^+^ cells. The same type of analysis was done in order to correlate Banff lesions with interstitial infiltrating cells but no clear associations were found (Supplementary Figure 7).

### Changes in the Inflammatory Infiltrates With Time

**Figure 8:**
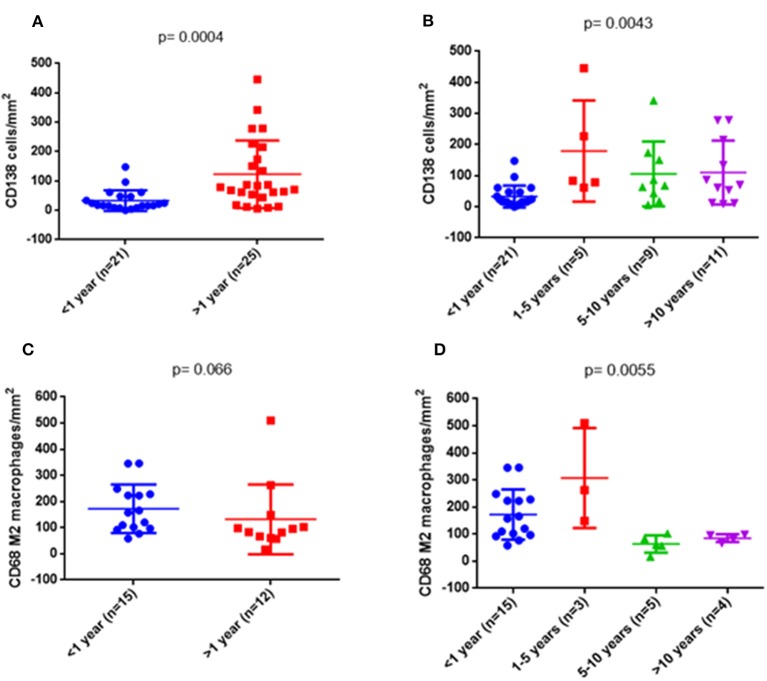
Quantitative changes of some cell types depending on the time of biopsy. On the left side, comparison was made between two groups: <1 year and >1 year. On the right side, the >1 year period was subdivided into three groups: 1–5, 5–10, and >10 years. Differences in plasma cells (*p* = 0.0004) **(A)** and a trend in M2 macrophages **(C)** were observed. On the right side, both cellular markers were significantly different **(B,D)**.

In order to see whether the changes in the composition of the infiltrates were time-dependent regardless of histological diagnoses, we analyzed the numbers of the interstitial infiltrating cells in different time periods. We started by considering only two periods, <1 and >1 year, and compared the values of each type of cells separately and the total number. There was a higher number of plasma cells (*p* = 0.0004) (Figure 8A) and a trend for a lower number of M2 macrophages (*p* = 0.066) in biopsies performed after the first year (Figure 8C). In a second approach, we divided the biopsies into four periods of time: <1, 1–5, 5–10, and >10 years. The increase in the number of plasma cells in biopsies at >1 year was confirmed, especially in the period 1–5 years (*p* = 0.0043) (Figure 8B). Differences in the amount of M2 macrophages became statistically significant (*p* = 0.0055) (Figure 8D). Neither the other cell types nor the global number of cells showed differences with regard to the period of time in which the biopsy was performed after the transplant (Supplementary Figure 8).

## Discussion

This is, to our knowledge, the first study that has examined the number of cells and the composition of the inflammatory infiltrates in several kidney rejection categories of the Banff classification by utilizing an innovative newCAST™-based approach. This method has the limitation that it requires many tissue samples, but it represents an objective tool that makes it possible to compare biopsies with different diagnoses regardless of the size of the tissue sample. The main advantage would be to support a correct diagnosis, which, in most cases, is challenging, but equally important is the opportunity to analyze *in situ* the players in the immune response and to understand the mechanisms of rejection.

In previous years, several authors have quantified lymphocytes or macrophages by different methods, mainly through counting cells in “high-power fields” (HPF), either visually (9) or automatically by utilizing Histoquest software-based analysis of digital pictures (10). Alternative methods to count cells by direct observation under a microscope with a 40x lens, either in the entire tissue or in selected areas of interest such as glomeruli, have been described (11, 12). More recently, the digitalization of scanned images has facilitated analyses with the use of different platforms. By computer-assisted analysis of graft inflammation in a group of AMR patients, Sicard et al. were able to define predictive factors for allograft loss such as the quantity of interstitial macrophages (13). Similarly, Bräsen et al. found that macrophage density in early surveillance biopsies had a predictive value for allograft loss but, counting macrophage density in early surveillance biopsies, would require (14), but it would require protocol biopsies, which are not performed in some hospitals. Our aim was to understand the events leading to rejection in each Banff category based on the knowledge of the cellular composition of the inflammatory infiltrates. We are conscious of the limitations of the present study, in which some of the rejection categories were not sufficiently represented. Nevertheless, we believe that this study has uncovered important findings. An ample number of markers of adaptive immunity, such as T and B lymphocytes and plasma cells, have been analyzed, but so also have markers of innate immunity such as macrophages and natural killer cells that have recently been investigated as having much more important roles in transplant rejection than previously thought (15, 16). In this sense, a very recent study by Yazdani et al., using transcriptomic data from AMR and TCMR patients, confirmed a role for NK cells in AMR by demonstrating significant enrichment of genes expressed in CD56^+^ NK cells in AMR. The main contribution of this study was that activated NK cells are the only cell type that discriminates between AMR and TCMR (17). Although our quantification study revealed that NK cells were almost undetectable and that the number of NK cells/mm^2^ was similar across all the rejection categories and the non-rejection group, principal component analysis revealed a remarkable clustering of aAMR and NK cells, which agrees with previous evidence for NK involvement in aAMR (15, 18). Moreover, a major innovation of this study was the characterization of macrophages, either non-polarized or polarized toward a proinflammatory state (M1) or a tissue-repair state (M2). As previously described, macrophages are able to switch their phenotype and function depending on the surrounding conditions. M1 macrophages are activated by signals provided by Th1 cells and are involved in chronic inflammation, while M2 macrophages are regulatory cells and produce anti-inflammatory cytokines (19). Based on our results, M1 macrophages clustered with BL, and we believe that the presence of such a high number of M1 macrophages could be a specific feature of BL that should be explored further. In addition, M2 cells may have a relevant role in MR, as these cells are found at higher levels in MR than in other rejection categories. Macrophage graft infiltration has been correlated with an increased incidence of chronic rejection (20, 21) as well as humoral rejection, as macrophage infiltration is significantly elevated in these categories (14). While aAMR cases and some cAMR cases have similar features, in this study, we found an important difference. T cells are the predominant cell type in cAMR, especially CD4^+^ cells, which were present in an amount double that of CD8^+^ cells, whereas macrophages are the predominant cell type in aAMR, supporting the results obtained by Magil in the glomeruli (12).

The main goal of the study was to understand the significance of the inflammatory infiltrates in biopsies. By using a computer-assisted stereology method, we determined immune profiles and found significant differences among the Banff rejection categories, principally regarding macrophage and T-cell populations. One of the main conclusions is that the absolute number of cells is crucial, but there is still an open question about what factors are able to alter the equilibrium of the cellular components in each case and drive the immune response to one form of rejection or another. In the coming years, these findings should be evaluated and replicated in a significantly higher number of patients in order to advance in the search for distinctive cellular profiles linked to different types of rejection.

## Data Availability Statement

The datasets generated for this study are available on request to the corresponding author.

## Ethics Statement

The studies involving human participants were reviewed and approved by Ethic committee of investigation of Hospitales Virgen del Rocio-Virgen Macarena, Sevilla. The patients/participants provided their written informed consent to participate in this study.

## Author Contributions

EA-D, RC-P, AS-B, AN-R, and IA participated in research design and selection of the patients. EA-D performed the experiments and the newCAST™ analysis. CA-M, AS-B, and IA provided additional laboratory data and contributed to the analysis. EA-D and IA wrote the manuscript. All authors approved the final version of the manuscript.

### Conflict of Interest

The authors declare that the research was conducted in the absence of any commercial or financial relationships that could be construed as a potential conflict of interest.

## References

[1] Solez K Axelsen RA Benediktsson H Burdick JF Cohen AH. International standardization of criteria for the histologic diagnosis of renal allograft rejection: the Banff working classification of kidney transplant pathology. Kidney Int. (1993) 44:11–422. 10.1038/ki.1993.259 8377384

[2] Saad R Gritsch HA Shapiro R Jordan M Vivas C Scantlebury V. Clinical significance of renal allograft biopsies with “borderline changes”, as defined in the Banff schema. Transplantation. (1997) 64:992–5. 10.1097/00007890-199710150-00010 9381547

[3] Haas M Loupy A Lefaucheur C Roufosse C Glotz D Seron D. The Banff 2017 kidney meeting report: revised diagnostic criteria for chronic active T cell–mediated rejection, antibody-mediated rejection, and prospects for integrative endpoints for next-generation clinical trials. Am J Transplant. (2018) 18:293–307. 10.1111/ajt.14625 29243394 PMC5817248

[4] Xu H Dhanireddy KK Kirk AD. Human monocytes as intermediaries between allogeneic endothelial cells and allospecific t cells: a role for direct scavenger receptor-mediated endothelial membrane uptake in the initiation of alloimmunity. J Immunol. (2006) 176:750–61. 10.4049/jimmunol.176.2.750 16393958

[5] Lawrence T Natoli G. Transcriptional regulation of macrophage polarization: enabling diversity with identity. Nat Rev Immunol. (2011) 11:750–61. 10.1038/nri3088 22025054

[6] Barros MH Hauck F Dreyer JH Kempkes B Niedobitek G. Macrophage polarisation: an immunohistochemical approach for identifying M1 and M2 macrophages. PLoS ONE. (2013) 8:e80908. 10.1371/journal.pone.0080908 24260507 PMC3829941

[7] Aguado-Domínguez E Gómez L Sousa JM Gómez-Bravo MA Núñez-Roldán A Aguilera I. Identification of the cellular components involved in *de novo* immune hepatitis: a quantitative immunohistochemical analysis. J Transl Med. (2018) 16:62. 10.1186/s12967-018-1440-8 29534755 PMC5851325

[8] Loupy A Haas M Solez K Racusen L Glotz D Seron D. The Banff 2015 kidney meeting report : current challenges in rejection classification and prospects for adopting molecular pathology. Am J Transplant. (2017) 17:28–41. 10.1111/ajt.14107 27862883 PMC5363228

[9] Fahim T Böhmig GA Exner M Huttary N Kerschner H Kandutsch S. The cellular lesion of humoral rejection: predominant recruitment of monocytes to peritubular and glomerular capillaries. Am J Transplant. (2007) 7: 385–93. 10.1111/j.1600-6143.2006.01634.x 17283488

[10] Bergler T Jung B Bourier F Kühne L Banas MC Rümmele P. Infiltration of macrophages correlates with severity of allograft rejection and outcome in human kidney transplantation. PLoS ONE. (2016) 11:e0156900. 10.1371/journal.pone.0156900 27285579 PMC4902310

[11] Moreso F Seron D O'Valle F Ibernon M Gomà M Hueso M. Immunephenotype of glomerular and interstitial infiltrating cells in protocol renal allograft biopsies and histological diagnosis. Am J Transplant. (2007) 7:2739–47. 10.1111/j.1600-6143.2007.02013.x 17949456

[12] Magil AB. Infiltrating cell types in transplant glomerulitis: relationship to peritubular capillary C4d deposition. Am J Kidney Dis. (2005) 45:1084–9. 10.1053/j.ajkd.2005.02.017 15957138

[13] Sicard A Meas-Yedid V Rabeyrin M Koenig A Ducreux S Dijoud F. Computer-assisted topological analysis of renal allograft inflammation adds to risk evaluation at diagnosis of humoral rejection. Kidney Int. (2017) 92:214–26. 10.1016/j.kint.2017.01.011 28318622

[14] Bräsen JH Khalifa A Schmitz J Dai W Einecke G Schwarz A. Macrophage density in early surveillance biopsies predicts future renal transplant function. Kidney Int. (2017) 92:479–89. 10.1016/j.kint.2017.01.029 28359537

[15] Hidalgo LG Sis B Sellares J Campbell PM Mengel M Einecke G. NK cell transcripts and NK cells in kidney biopsies from patients with donor-specific antibodies: evidence for NK cell involvement in antibody-mediated rejection. Am J Transplant. (2010) 10:1812–22. 10.1111/j.1600-6143.2010.03201.x 20659089

[16] Hirohashi T Chase CM Della Pelle P Sebastian D Alessandrini A Madsen JC. A novel pathway of chronic allograft rejection mediated by NK cells and alloantibody. Am J Transplant. (2012) 12:313–21. 10.1111/j.1600-6143.2011.03836.x 22070565 PMC3667648

[17] Yazdani S Callemeyn J Gazut S Lerut E Loor H Wevers M. Natural killer cell infiltration is discriminative for antibody-mediated rejection and predicts outcome after kidney transplantation. Kidney Int. (2019) 95:188–98. 10.1016/j.kint.2018.08.027 30396694

[18] Venner JM Hidalgo LG Famulski KS Chang J Halloran PF. The molecular landscape of antibody-mediated kidney transplant rejection: evidence for NK involvement through CD16a Fc receptors. Am J Transplant. (2015) 15:1336–48. 10.1111/ajt.13115 25787894

[19] Mosser DM Edwards JP. Exploring the full spectrum of macrophage activation. Nat Rev Immunol. (2008) 8:958–69. 10.1038/nri2448 19029990 PMC2724991

[20] Mannon RB Macrophages: contributors to allograft dysfunction, repair, or innocent bystanders? Curr Opin Organ Transplant. (2012) 17:20–5. 10.1097/MOT.0b013e32834ee5b6 22157320 PMC3319132

[21] Toki D Zhang W Hor KL Liuwantara D Alexander SI Yi Z. The role of macrophages in the development of human renal allograft fibrosis in the first year after transplantation. Am J Transplant. (2014) 14:2126–36. 10.1111/ajt.12803 25307039

