# Supplementary material for: Computer-Assisted Definition of the Inflammatory Infiltrates in Patients With Different Categories of Banff Kidney Allograft Rejection
Source: Front Immunol. 2019 Nov 8;10:2605. doi: 10.3389/fimmu.2019.02605 (PMC6856956; doi:10.3389/fimmu.2019.02605)
Supplement: Supplementary file 1 [file Data_Sheet_1.docx]

**Supplementary Table 1.** Antibody-conditions for immunohistochemistry and immunofluorescense experiments.

| Target | Supplier | Reference | Host specie | Clone | Dilution | Incubation time | Incubation temperature |
| --- | --- | --- | --- | --- | --- | --- | --- |
| Anti-CD20 | Dako | N1502 | rabbit | polyclonal | RTU | 1h | RT |
| Anti-CD138 | Dako | M7228 | mouse | MI15 | 1:80 | o/n | 4°C |
| Anti-CD4 | Abcam | ab846 | mouse | IF6 | 1:4 | o/n | 4°C |
| Anti-CD8 | Abcam | ab93278 | rabbit | EP1150Y | 1:500 | 2h | RT |
| Anti-CD56 | Monosan | MON9006-1 | mouse | 123C3 | 1:20 | o/n | 4°C |
| Anti-FOXP3 | Abcam | ab20034 | mouse | 236A/E7 | 1:50 | 2h | RT |
| Anti-CD68 | Santa Cruz Biotechnology | sc-70761 | mouse | 3F103 | RTU | 1h | RT |
| Anti-pSTAT1 | Santa Cruz Biotechnology | sc-135648 | rabbit | polyclonal | 1:20 | 1h | RT |
| Anti-cMAF | Santa Cruz Biotechnology | sc-7866 | rabbit | polyclonal | 1:40 | 1h | RT |

RTU: ready to use; RT: room temperature; o/n: overnight.

**Supplementary Table 2. Cell count in biopsy at the time of diagnosis. Only the patients with all the markers completed are included.**

| **Patient code** | **CD138** | **CD20** | **CD4** | **CD8** | **CD68 non-P** | **M1 ϕ** | **M2 ϕ** | **CD56** | **FoxP3** | **Total # cells/mm^2^** |
| --- | --- | --- | --- | --- | --- | --- | --- | --- | --- | --- |
| **NR-1** | 16 | 2 | 209 | 70 | 338 | 43 | 77 | 3 | 5 | 763 |
| **NR-2** | 8 | 3 | 45 | 28 | 461 | 62 | 97 | 3 | 0 | 707 |
| **NR-3** | 16 | 54 | 206 | 113 | 305 | 38 | 158 | 2 | 15 | 907 |
|  |  |  |  |  |  |  |  |  |  |  |
| **aAMR-1** | 9 | 2 | 107 | 64 | 487 | 11 | 92 | 0 | 4 | 774 |
| **aAMR-2** | 15 | 7 | 195 | 30 | 182 | 9 | 120 | 30 | 8 | 596 |
| **aAMR-3** | 44 | 228 | 342 | 408 | 153 | 27 | 17 | 2 | 26 | 1 245 |
| **aAMR-4** | 34 | 213 | 117 | 480 | 516 | 39 | 223 | 15 | 47 | 1 685 |
|  |  |  |  |  |  |  |  |  |  |  |
| **cAMR-1** | 87 | 26 | 284 | 205 | 274 | 90 | 95 | 3 | 3 | 1 066 |
| **cAMR-2** | 6 | 9 | 135 | 44 | 466 | 161 | 102 | 3 | 0 | 926 |
| **cAMR-3** | 151 | 75 | 444 | 335 | 359 | 37 | 61 | 1 | 7 | 1 469 |
| **cAMR-4** | 279 | 102 | 1 284 | 348 | 280 | 13 | 67 | 12 | 116 | 2 501 |
|  |  |  |  |  |  |  |  |  |  |  |
| **BL-1** | 174 | 176 | 1467 | 797 | 698 | 63 | 56 | 2 | 44 | 3 477 |
| **BL-2** | 4 | 164 | 656 | 283 | 361 | 23 | 58 | 1 | 32 | 1 582 |
| **BL-3** | 21 | 307 | 368 | 218 | 537 | 144 | 223 | 2 | 13 | 1 834 |
| **BL-4** | 227 | 332 | 286 | 1012 | 924 | 250 | 262 | 2 | 68 | 3 363 |
| **BL-5** | 24 | 128 | 812 | 144 | 972 | 249 | 166 | 0 | 46 | 2 541 |
| **BL-6** | 18 | 20 | 686 | 187 | 766 | 338 | 228 | 2 | 43 | 2 286 |
|  |  |  |  |  |  |  |  |  |  |  |
| **MR-1** | 446 | 164 | 739 | 667 | 1 604 | 45 | 510 | 18 | 332 | 4 525 |
| **MR-2** | 25 | 115 | 850 | 1 112 | 1 419 | 39 | 345 | 8 | 48 | 3 961 |
| **MR-3** | 278 | 252 | 1 815 | 1 325 | 813 | 96 | 98 | 4 | 101 | 4 782 |
| **MR-4** | 15 | 355 | 1 402 | 375 | 893 | 31 | 249 | 19 | 108 | 3 447 |

NR, nonrejection; aAMR, active antibody-mediated rejection; cAMR, chronic active antibody-mediated rejection; BL, Borderline; MR, mix rejection.


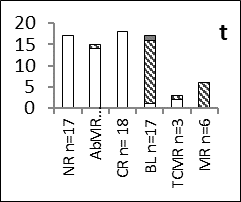

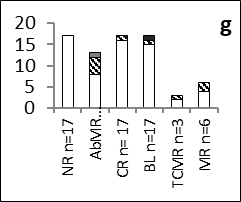

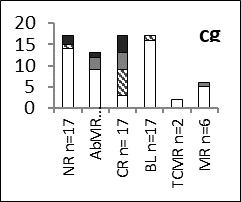

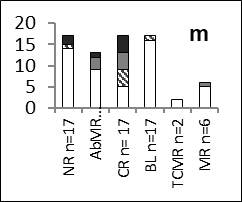

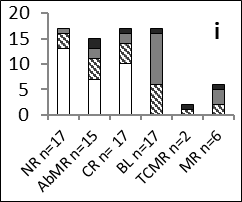

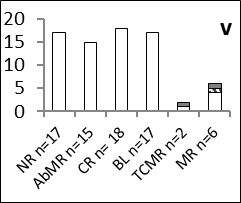

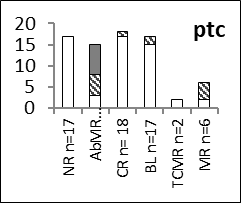

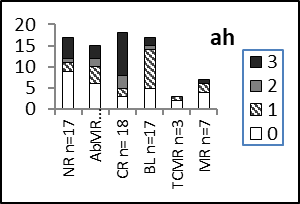

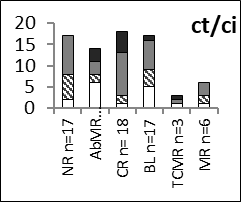

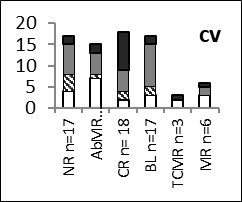

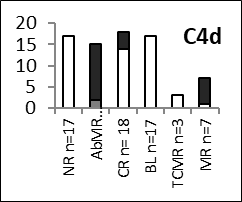


**Supplementary Figure 1.** Banff scores of all biopsies studied. Number of patients is specified in every rejection group.

g, glomerulitis; cg, allograft glomerulopathy; mm, mesangial matrix increase; cv, vascular fibrous intimal thickening; ct, tubular atrophy; ci, interstitial fibrosis; t, tubulitis; i, interstitial inflammation; C4d; v, intimal arteritis; ptc, peritubular capillaritis; ah, arteriolar hyaline thickening; IFTA, interstitial fibrosis and tubular atrophy. Scores are 0, 1, 2, and 3. IFTA and glomerular percentage are represented in %.


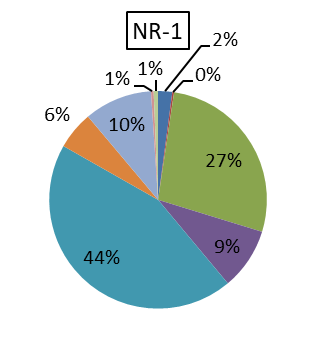

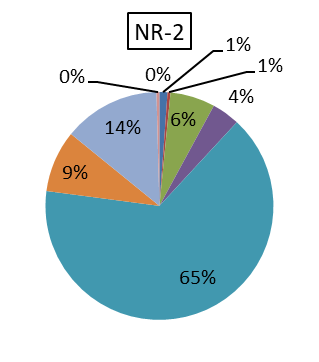

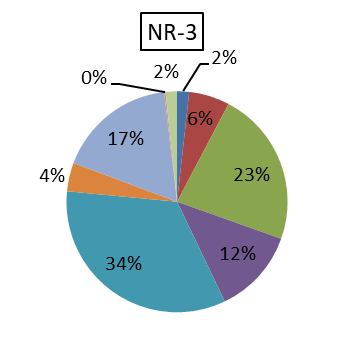

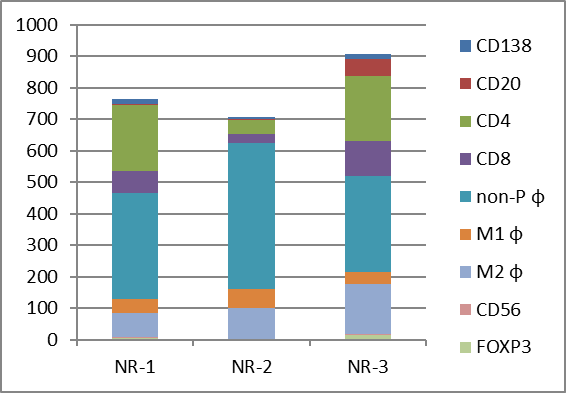


**Supplementary Figure 2.** Detailed description of the infiltrates found in three patients of the nonrejection group (NR).


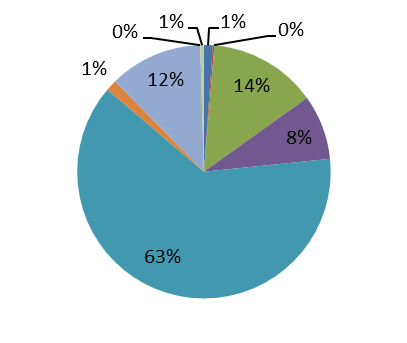

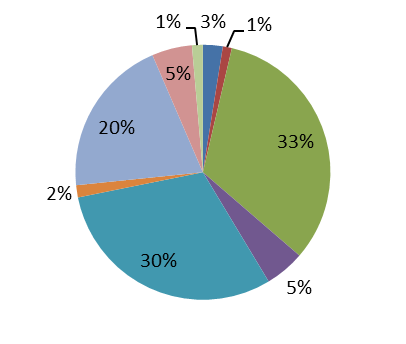

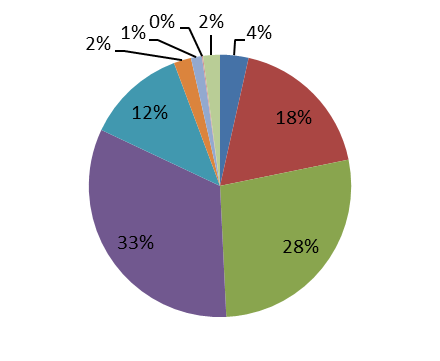

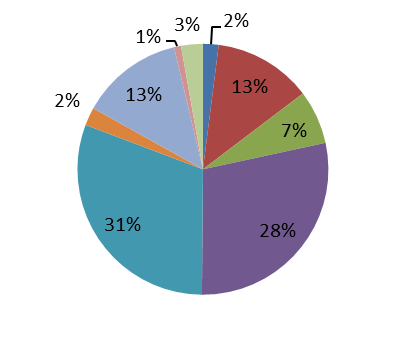


aAMR-1

aAMR-3

aAMR-2

aAMR-4

**Supplementary Figure 3.** Detailed description of the infiltrates found in 4 patients of the Banff active antibody-mediated rejection (aAMR) group.


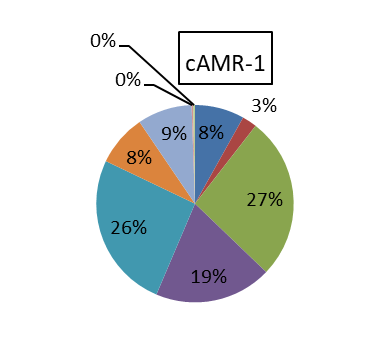

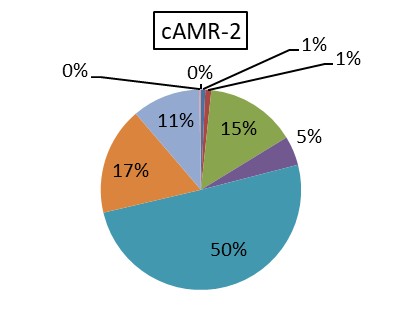

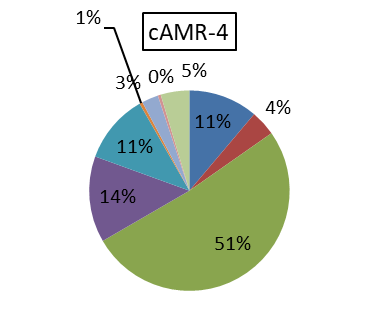

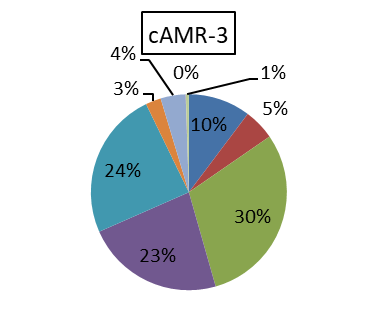

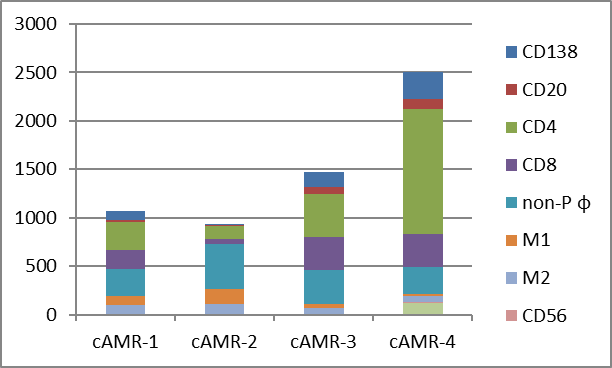


**Supplementary Figure 4.** Detailed description of the infiltrates found in 4 patients of the Banff chronic active antibody-mediated rejection (cAMR) group.


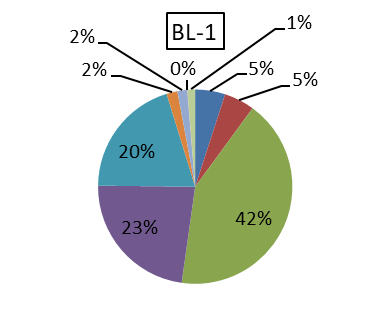

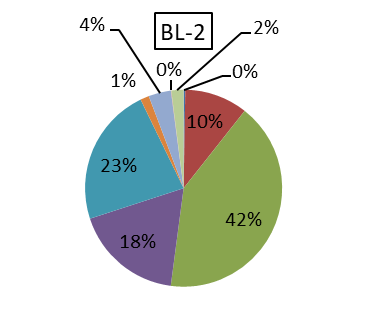

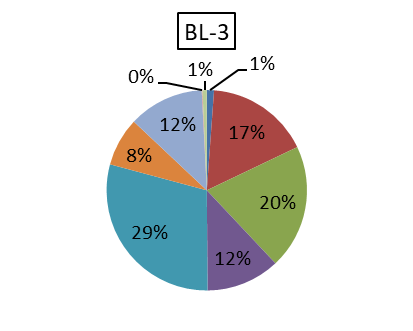

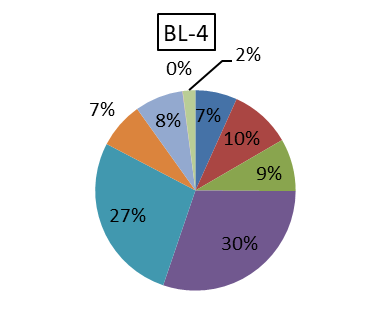

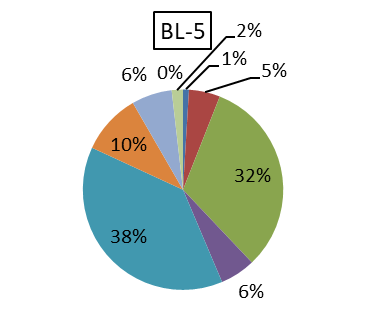

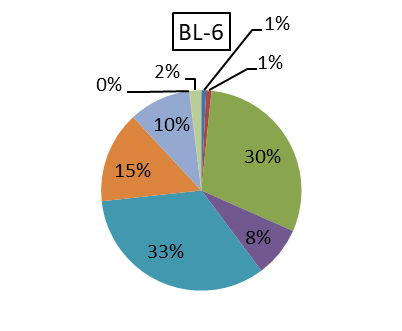

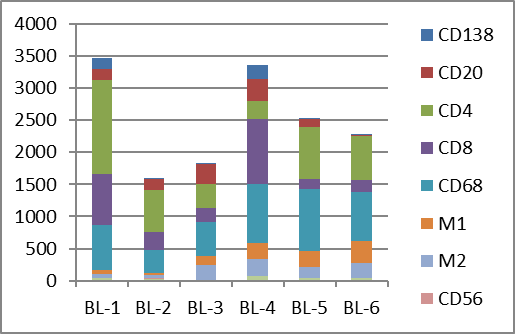


**Supplementary Figure 5.** Detailed description of the infiltrates found in 6 patients of the Banff borderline (BL) group.


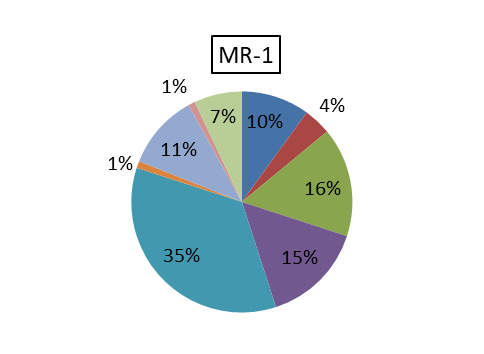

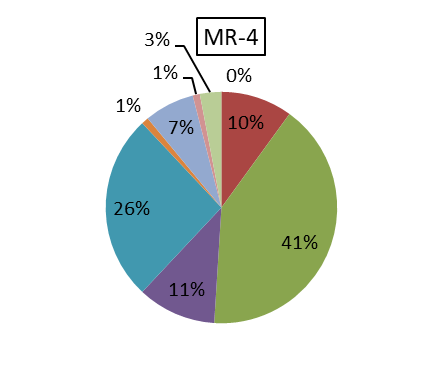

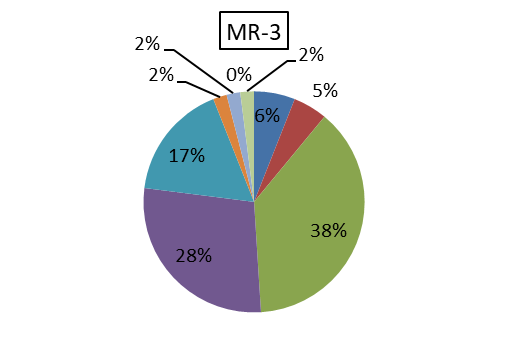

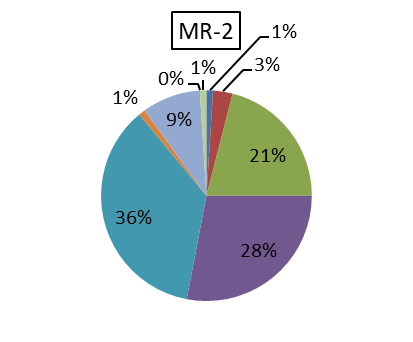

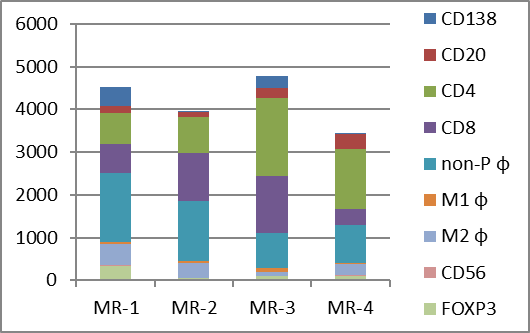


**Supplementary Figure 6.** Detailed description of the infiltrates found in 4 patients of the Banff mixed rejection (MR) group.


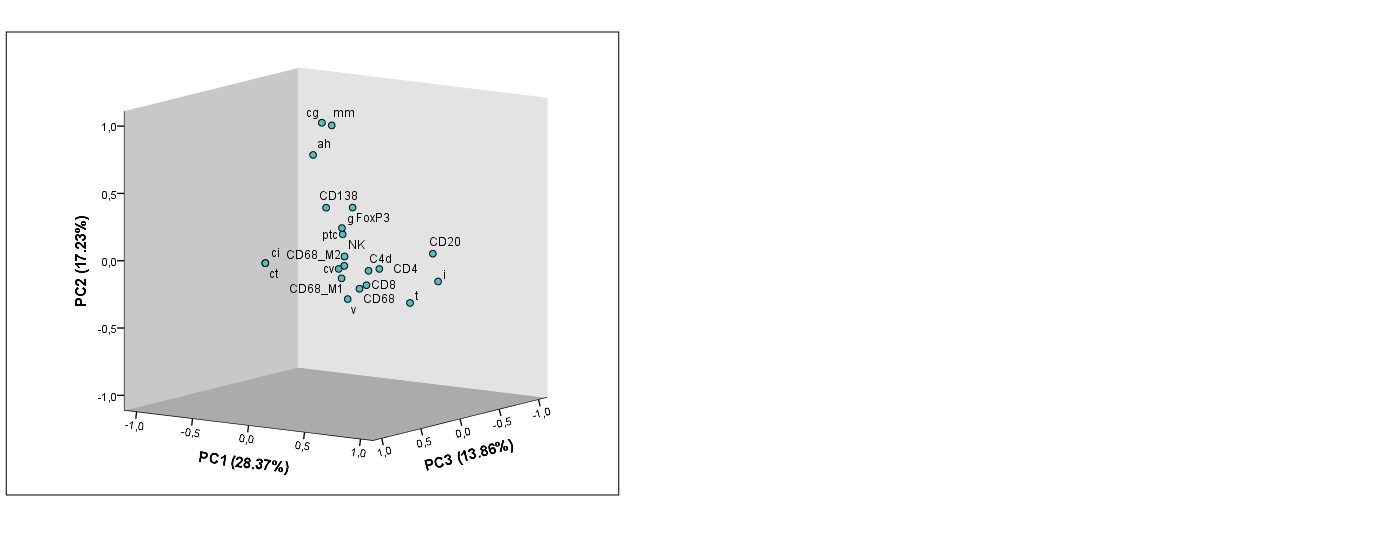


**Supplementary Figure 7.** Principal component analysis (PCA) scatterplot of semiquantitative scores (0, 1, 2, 3) obtained for each Banff lesion (g, i, t, v, cg, ci, ct, cv, mm, ptc, C4d) and cellular types of the inflammatory infiltrates.


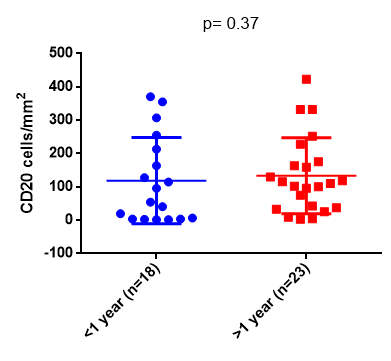

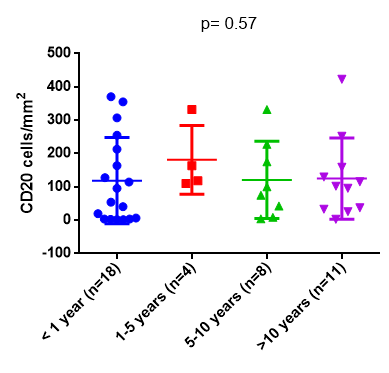

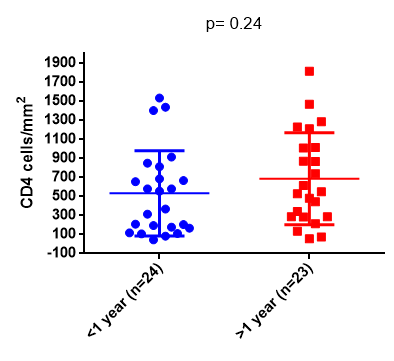

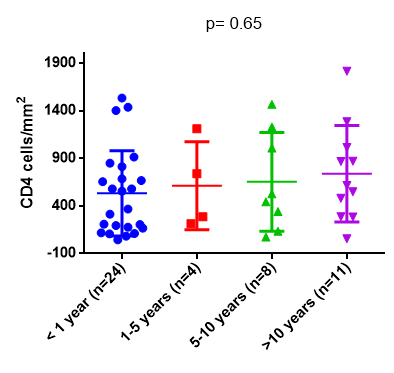

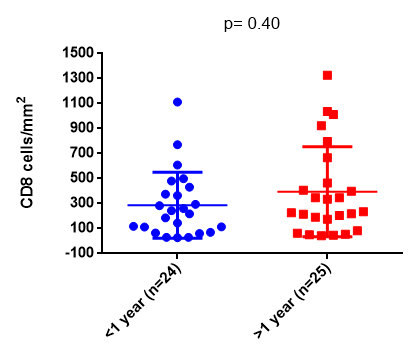

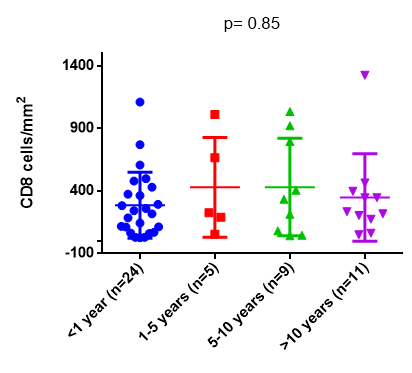

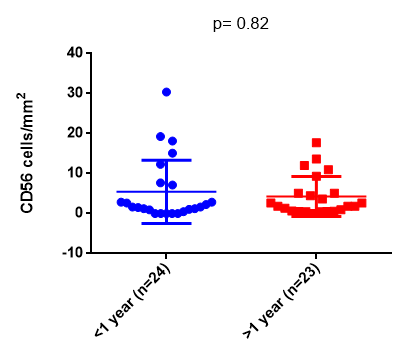

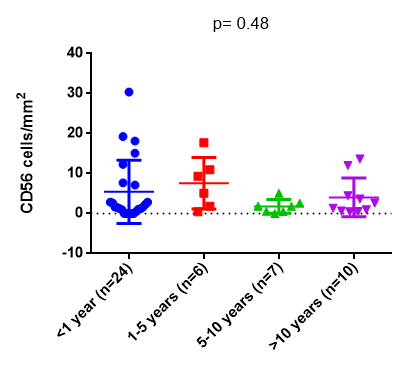

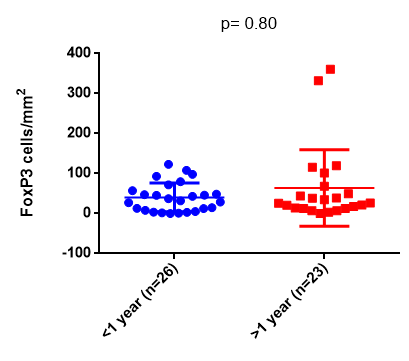

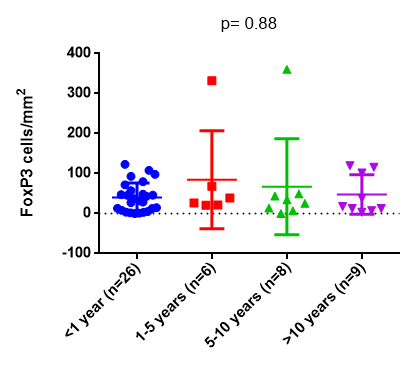

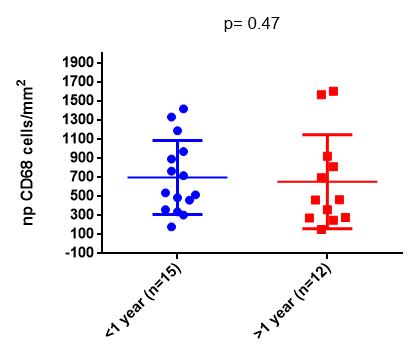

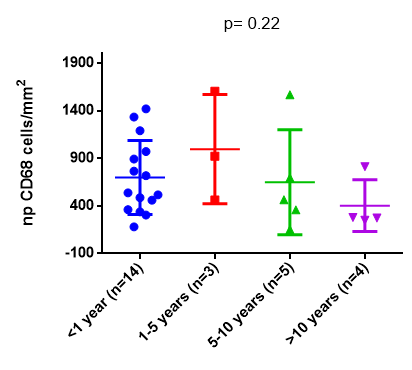

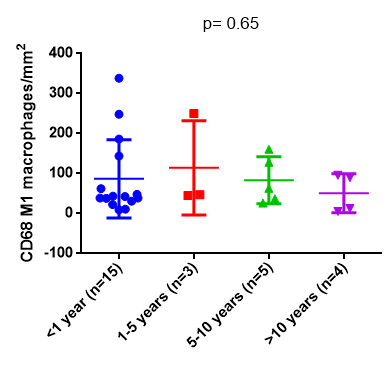

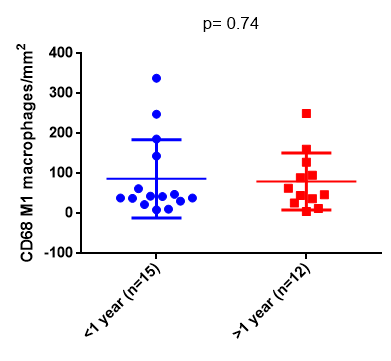

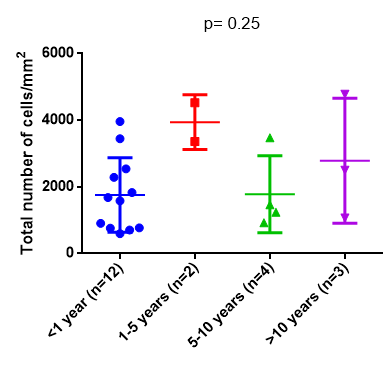

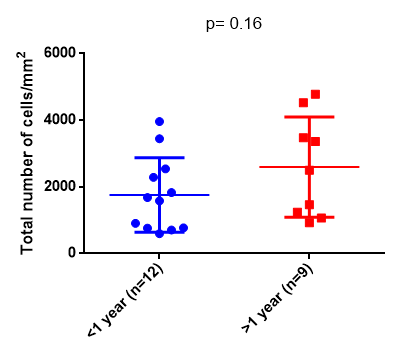


different.

**Supplementary Figure 8.** Comparison of the changes in the composition of the infiltrates in biopsies, taking into consideration timing of biopsy. On the left column the biopsies have been distributed in 2 periods: <1 year and > 1 year. On the right column the biopsies have been distributed in 4 periods: <1 year, 1-5 years, 5-10 years and >10 years. The composition of these cellular types is not statistically different.
